# Consistency of Medical Data Using Intelligent Neuron Faster R-CNN Algorithm for Smart Health Care Application

**DOI:** 10.3390/healthcare8020185

**Published:** 2020-06-25

**Authors:** Seong-Kyu Kim, Jun-Ho Huh

**Affiliations:** 1 Department of Information Technology, Sungkyunkwan University, Seoul 03063, Korea; guitara7@skku.edu; 2 Department of Data Informatics, Korea Maritime and Ocean University, Busan 49112, Korea

**Keywords:** Intelligent agent, neuron computer, health care system, electronic medical record, cloud architecture, artificial intelligence

## Abstract

The purpose of this study is to increase interest in health as human life is extended in modern society. Hence, many people in hospitals produce much medical data (EMR, PACS, OCS, EHR, MRI, X-ray) after treatment. Medical data are stored as structured and unstructured data. However, many medical data are causing errors, omissions and mistakes in the process of reading. This behavior is very important in dealing with human life and sometimes leads to medical accidents due to physician errors. Therefore, this research is conducted through the CNN intelligent agent cloud architecture to verify errors in reading existing medical image data. To reduce the error rule when reading medical image data, a faster R-CNN intelligent agent cloud architecture is proposed. It shows the result of increasing errors of existing error reading by more than 1.4 times (140%). In particular, it is an algorithm that analyses data stored by actual existing medical data through Conv feature map using deep ConvNet and ROI Projection. The data were verified using about 120,000 databases. It uses data to examine human lungs. In addition, the experimental environment established an environment that can handle GPU’s high performance and NVIDIA SLI multi-OS and multiple Quadro GPUs were used. In this experiment, the verification data composition was verified and randomly extracted from about 120,000 medical records and the similarity compared to the original data were measured by comparing about 40% of the extracted images. Finally, we want to reduce and verify the error rate of medical data reading.

## 1. Introduction

**Figure 1:**
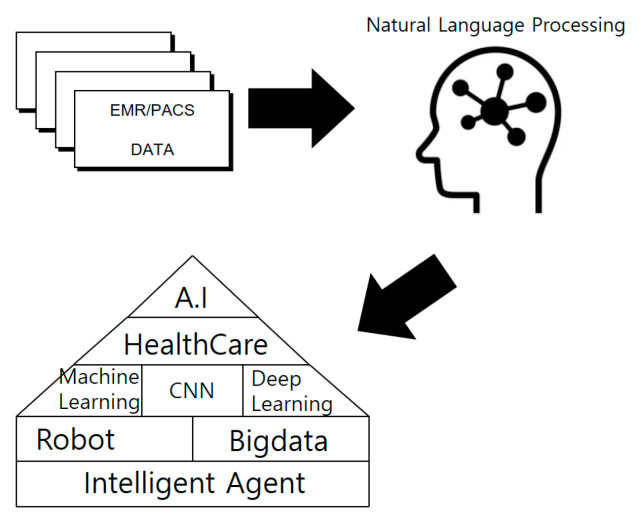
Schematic diagram of health care artificial intelligence.

In modern society, human life is much longer than it used to be. Many medical accidents are happening in countries where health care is not provided. These reasons led to the study of verification of accurate reading of medical imaging data [1]. After all, people all over the world are very interested in health. Therefore, many people take care of the hospital and then many medical data (EMR, PACS, OCS, EHR, MRI, X-ray) are produced. Moreover, these medical data are stored as structured and unstructured data. However, many medical data are causing errors, omissions and mistakes in the process of reading. These actions are very important in dealing with human life and sometimes lead to medical accidents due to doctor’s mistakes. Therefore, a study is conducted to verify errors in reading existing medical image data through the CNN intelligent agent cloud architecture. In addition to reduce the error rule when reading medical image data a faster R-CNN intelligent agent cloud architecture is proposed (See Figure 1).

We use a method to reduce errors in existing error reading. In particular, an algorithm was applied to analyze data stored by actual existing medical data through Conv feature map using deep ConvNet and ROI projection. The data were screened using about 120,000 databases. We used data to examine the lungs of the chest. In addition, the experimental environment established an environment that can handle high performance GPUs. NVIDIA SLI multi-OS and multiple Quadro GPUs were used. In this experiment, the verification data composition were verified and randomly extracted from about 120,000 medical records, and the similarity compared to the original data were measured by comparing about 40% of the extracted images. Most of these images were acquired from public health data. Therefore, the purpose is to verify these using public data. Finally, the artificial intelligence system could not be a device that can solve everything. However, we want to reduce the error rate of reading medical data and verify that the medical accident rate is unfamiliar and accumulated by verifying similarity of medical data in artificial intelligence [2,3,4].

## 2. Background Knowledge

### 2.1. Artificial Intelligence

Artificial intelligence is a technology that combines human learning ability, reasoning ability, perceptual ability and understanding of natural language through computer programs. As a branch of computer science and information technology that studies how computers can make thinking, learning and self-development possible with human intelligence, artificial intelligence allows computers to emulate human intelligent behavior [5,6,7]. This concept of artistic intelligence is already used in many areas. In addition, artificial intelligence is widely used in the medical system [8,9,10,11]. However, with artificial intelligence facing several crises, verification algorithms are now being used to do many of the necessary research. Algorithms used in the medical system are already used for many kinds of surgery, reading, etc. In fact, artificial intelligence robots are used in China to assist in surgery [12,13,14,15,16].

**Figure 2:**
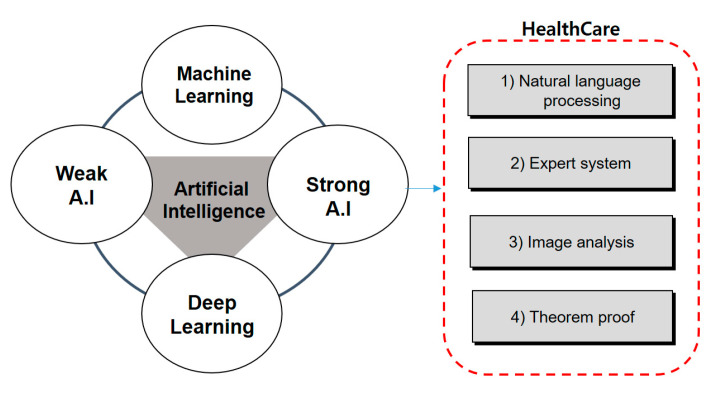
Relationship between artificial intelligence and health care.

In addition, such artificial intelligence is evolving from early artificial intelligence systems to machine learning and deep learning. This artificial intelligence technology is also being used in the medical field (See Figure 2).

(1). Natural Language Processing

Natural language processing already makes systems such as automatic translation practical. When further research is conducted, we expect people will be able to talk to computers and exchange information, which will bring about innovative changes in their use.

(2). Expert system

Expert systems allow computers to replace the various professional tasks humans are currently doing (such as diagnosing by doctors, assessing mineral reserves, estimating the structure of the compound and judging damage compensation premiums). It was also the earliest development among various fields.

(3). Video and Image Analysis

Video and image analysis is very complicated and impossible without the introduction of artificial intelligence theory, such as analyzing a video that a computer captured through a TV camera to find out what it is or to hear a person’s voice and convert it into a sentence. These video and voice recognition tasks are key technologies for character recognition, robotics, etc.

(4). Theorem Proof

Theorem proof is an essential technology used in various fields of artificial intelligence as a process of logically deducing and proving mathematical theorems from already known facts. It of itself has much value.

(5). Neural Networking

Neural Networking is relatively recent, and is not a mathematical logic, but rather a neural network structure consisting of a network of numerous simple processors, imitating the human brain.

#### 2.1.1. Weak Artificial Intelligence

**Figure 3:**
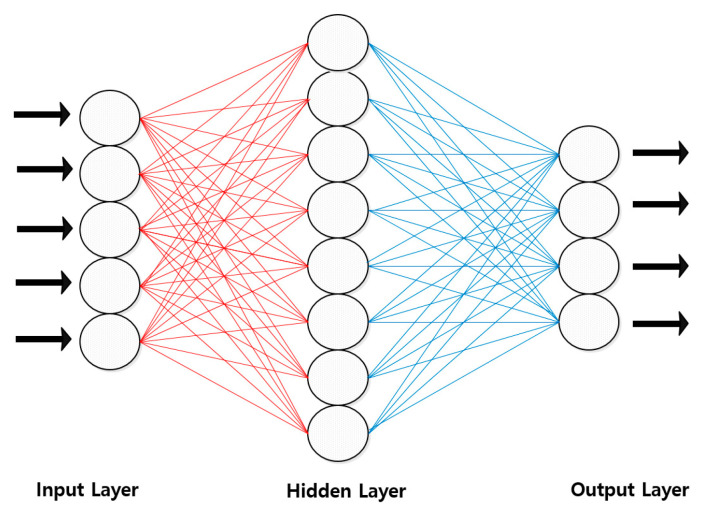
Weak artificial intelligence.

Weak artificial intelligence focuses on making computers perform various problems that humans can easily solve, such as finding objects in medical data such as MRIs and X-rays, listening to voice sounds and understanding the situation. It is artificial intelligence that is being developed with more practical goals, rather than aiming for the much more vague human intelligence. It tends to be used as a tool to solve certain problems rather than something with intelligence. Based on the above definition, all artificial intelligence created by humans so far is about artificial intelligence [17,18,19]. This is a predefined algorithm for medical data analysis and verification a level that enables people to make relatively intelligent behavior or decisions based on vast amounts of data. Even though artificial intelligence can find rules and solve problems on its own, it is hard to know why it solved them. A problem can only be solved within a limited range. Hence, it is very difficult to verify medical data. Medical artificial intelligence systems are already far beyond human capabilities with functions they can do, such as surgical data and examination data. There are many things that humans cannot do among these artificial intelligence tasks [20]. It is not human because it did not imitate humans. Instead, it was able to transcend humans. The ability to simply solve a given problem can be superior to the ability of strong artificial intelligence. This is called medical reinforcement learning. This is somewhat predictable: even if strong artificial intelligence has yet to appear, as one cannot say “yes” to the question of “Does human intelligence have outstanding advantages in solving any problems?” For sure (see Figure 3).

#### 2.1.2. Strong Artificial Intelligence

Strong artificial intelligence is implemented by a computer’s ability to process information of medical data. It is also a computer-generated file that scans the entire brain, whether it is a program with the same intelligence as a human through a medical system. Of course, this means that even a single human being has a level of intellectual ability which is why most of the controversial artificial intelligence problems [21] are caused by artificial intelligence. Artificial intelligence which can be called medical strong artificial intelligence is being developed for medical data analysis [22,23,24,25]. This is because human judgment cannot control human life. Medical machines and computers need to be able to do some work to be considered equal to humans [26]. Even if medical devices may be able to do a similar level of work in the future, there are many barriers to the creation of strong engineering. For example, how do doctors in a medical field’s consciousness, mind and thoughts still occur in unknown areas, and the doctor who is the party may not know whether it exists physically or whether it can give consciousness to an artificial object other than the brain. In this regard, there is a mental and physical problem, whether the mind, which can be viewed with hardware, can be separated from each other or not. There is a dualism in which people view each other as separate beings—such as the body and the soul and the monism is called monism [27,28].

**Figure 4:**
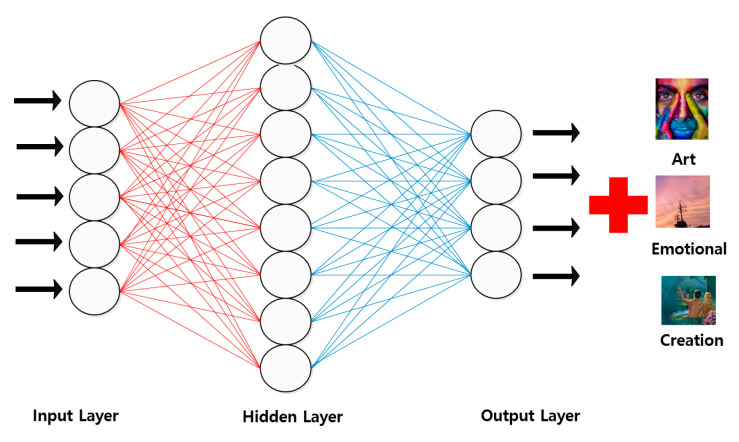
Strong artificial intelligence.

Medical data are one of the most important forms of data. If these data are misread, or there is a problem, it may eventually lead to death. Therefore, this study is studied by strong artificial intelligence (see Figure 4).

### 2.2. Intelligent Agents

A medical intelligent agent means that all programs act on behalf of a person. This allows all behaviors and patterns of doctors who can perform medical surgery to be called agents. On the other hand, there is the interpretation that computer programs can never be agents in terms of not being able to do as much work as people want. The characteristics of medical intelligent agents refer to what doctors think. Their features include doctors’ autonomy, sociality, mobility and intelligence. Here, the autonomy of wizards refers to the ability of agents to make their own judgments and work without direct instruction or interference from users or other programs. This is the most important feature of a medical geodetic agent. In addition, agent mobility can be very useful even if an agent performing tasks is moved to a server system in an environment where it is difficult to maintain continuous contact with mobile computers and server systems. This can occur in an environment where these medical data are performed, and later the agent is brought to its own system to receive results. It refers to the ability to move to other medical computer systems and perform tasks. In addition, the managed agent needs help from agents and others to analyze inputs to determine the user’s intentions. Thus, when multiple agents work together, they can provide more services to users compared to when they perform independently. Intelligence is the ultimate goal in the field of computers that all medical programs want to have [28,29,30].

**Figure 5:**
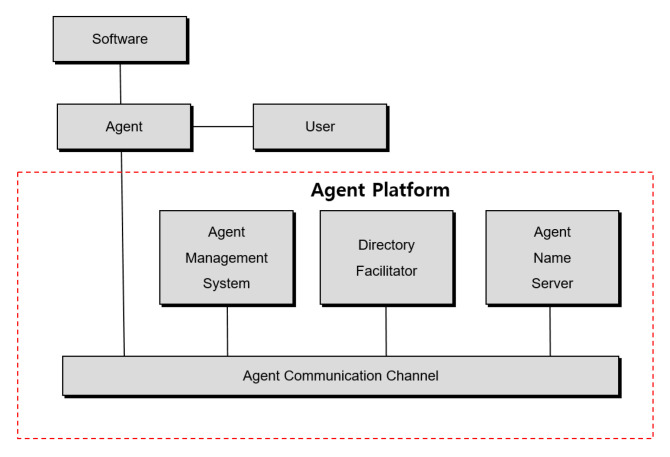
Intelligent platform agents.

But intelligence is an essential element for intelligent agents. In order for an agent to have autonomy, it must have the ability to judge according to each situation. To perform tasks through cooperation with other agents, it must have the ability to process knowledge related to task planning, division and integration of performance results. To move to other systems and process tasks, it is necessary to have the ability to judge the server to move. Intelligent agents also learn the characteristics of users to provide them with a more convenient computer experience. It is necessary to have the ability to deduce specific tasks for vague requirements or to accumulate new experiences from past work. In addition to these basic characteristics, some of the features that an agent should have include responsiveness, good conduct, honesty and rational behavior. Of course, agents must always have all of these features. However, those who study agents believe that the more satisfied the program is with these features, the closer it becomes to a more complete agent. From the perspective of artificial intelligence, intelligent agents want to play the role of searching and automating using active agents from the perspective of health care (see Figure 5).

### 2.3. AI and Health Care

Artificial intelligence is divided into areas previously referred to as “intelligent agents” and today’s ‘machine learning, deep learning and self-learning,’ where many researchers conducted research on the subject [31,32]. This study analyses the extraction of image data, such as EMR, PACS, MRIs and X-rays, which are widely used in these medical treatments. In addition, the distinctive feature of AI is its “self-learning” ability to enhance its own performance. AI was initially learning how the brain works, but it could eventually describe its principles. Early artificial intelligence health care developed an algorithm called ‘temporary difference learning’, which after receiving ‘temporary difference learning’ estimates how close compensation time is and changes the work process each time [33] instead of changing the entire work process depending on the reward. In addition, we should always pay great attention to technology development and security, considering the number of cases where image data is falsified or misplaced by hackers in the middle. Artificial intelligence cannot change all medical systems, but it seekd to at least play a role in significantly reducing medical accidents [34,35,36,37,38].

### 2.4. Smart Health Care

Smart health care is a new concept that combines health care, AI, big data, Internet of Things, cloud and nanotechnologies. It is a field that has recently drawn attention as individuals can easily receive health care anytime, anywhere. The health care sector has evolved from traditional hospital-focused medical industry areas to combine ICTs with the medical sector to provide more convenient and diverse forms of health-related services to diverse consumers. In particular, policy, technical and social response strategies to these changes are needed as digital technologies are rapidly developing outside the medical sector as artificial intelligence, Internet of Things and wearable devices have recently been incorporated into the medical sector [39]. The health care sector has been developed by reflecting market demand to address soaring medical expenses due to aging populations and increases in chronic diseases, as the medical care industry has changed significantly from past treatment with innovation in various ICT convergence technologies and bio sectors to prevent diseases and to cater to consumers who manage their own daily lives. There have been various definitions made recently amid the interest in smart health care; a major key point of the concept is to expand the existing health care industry by combining ICT and medical technologies. Smart health care is moving further from the center of medical products through a platform that integrates various data and services associated with personal health care, ultimately evolving in the direction of intelligent health care solutions that evolve a number of systems through organic combinations in real time. The smart health care industry is an industry where smart device technology and information and communication technology converge with health care and medical services and it is being developed with three major types: hardware, software and services (see Table 1).

Rapidly evolving trends in IT and convergence technologies in particular further increase the importance of technology standards. In the past, a technology innovation might have been an individual medical device; now, it is time for global standard competitiveness for a global industry to be competitive, as it has to work with various systems and maximize usability. Therefore, this report seeks to select the areas of focus that are expected to be promoted first among smart health care convergence products that are rapidly developing at this point in time and to analyze key related trends while drawing out areas that need to be addressed from the technical, standard and policy perspectives to enable strategic establishment with a large picture of related technologies, standards and policies. In the present era as income growth and advances in medical technology improve the quality of life health approaches and changes in thinking and definition of life as well as existing treatment-focused medical paradigms are shifting to predictive, proactive, personalized and participatory. Smart health care is seen as an effective solution and an alternative, especially at a time when the fast-paced aging population is increasing medical spending and the demand for medical care for chronic patients continues to grow [40]. Therefore, in this study, we present a methodology for verifying individual medical information in the right place using these health care and artificial intelligence intelligent agents [41].

## 3. Smart Health Care Application Architecture with Intelligent Agent

### 3.1. Issue Raising

To improve health care services using intelligent agents, existing smart health care needs to be analyzed. Smart health care is undoubtedly a promising industry to which the world is paying attention. Still, due to regulations, it is difficult for the industry to grow in Korea. As obstacles to growth in the smart health care sector in Korea, first, there is too much unshared medical information. Health information alone, which is not combined with medical information, will inevitably have a limited number of services to offer. Under the current law, it is not possible to store medical information in the cloud. Instead, medical information must be stored on servers within a medical institution and may not be linked to external systems. On the other hand, in the US, services and ecosystems using cloud-based medical information used by doctors are being rapidly established. For example, a health care service approved by the US FDA (Food and drug administration) in conjunction with a private insurance company based on the consent of the patient stores patient medical information and health information in the cloud and sends it to the hospital. Upon receiving the transmitted data, a medical institution can give a prescription to a patient based on the health management data. Japan whose medical environment is similar to that of Korea has earlier begun legal revisions to incorporate the cloud into its health care industry; thus, increasing its cloud-based health care services. In China, the introduction of cloud services in the medical–health care sector is ongoing, although it is still in the early stages. In addition, medical data (EMR, PACS, MRI, PHR, X-ray, etc.) lacks some reading and verification, leading to medical accidents. Due to regulations, in the smart health care industry, which utilizes connected medical devices and wearable terminals for medical services, Korean companies will inevitably lag behind in competing with global companies. In addition, in the absence of criteria for anonymous medical information for big data analysis, medical information is defined as sensitive information among personal information, so separate consent from the information provider is essential to take advantage of it [42].

Nonetheless, the personal information protection act allows the use of information for research purposes because it allows the use of medical big data of certain individuals if provided in unrecognizable form for the purpose of preparing statistics and academic research. Still, the current privacy law does not separate individual identifiers from the health information needed for research, so the scope of the information that requires anonymity is also unclear. This study aimed at using intelligent agents of artificial intelligence to activate this health care system. The goal is to complete an artificial intelligence system in which our personal information is automatically collected using intelligent agents and such personal information is automatically analyzed and delivered using smart devices. To date, however, such artificial intelligence services need to be collected through an integrated engine with various health care services. Based on these collected data, intelligent agents collect based on engine and use these agents to create a standard architecture. This study seeks to establish a standardization architecture of intelligent agents for these unstandardized artificial intelligence algorithms and for deep learning the necessary health information [43].

### 3.2. Research Methodology

Using intelligent agents, EMR data are structured and quantified like blood count tests, but there are many different types of data such as natural language, medical abbreviations and unstructured data when medical staff describe medical records in sentences. Prior to deep learning, experts with medical knowledge had to set up critical variables directly to create EMR data-based predictive models and consume 80% of project resources in the course of preprocessing the data. The performance of the predictive model was heavily influenced by the expert’s prior knowledge of preparing learning data; above all, there were limitations wherein EMR data could not be utilized and only some data could be used. Deep learning allows we to learn the entire EMR data that was previously impossible to learn, such as medical notes made up of natural language.

**Figure 6:**
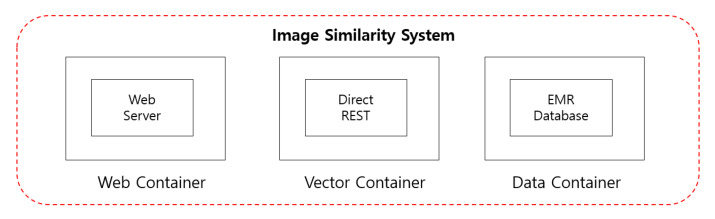
Image similarity system for extracting EMR data.

In addition, deep learning for medical data requires designing characteristics and important variables of data in advance to learn medical data in artificial intelligence, but deep-learning designs are designed with algorithms that self-discover and calculate which variables or combinations are important. It also presents an intelligent methodology that eliminates the need for 80% of preprocessing medical data. It also preprocesses more data to increase the accuracy of models that add new kinds of data to intelligent artificial intelligence models or verify medical data for easier application to other hospitals or sites. In this study, we introduce the methodology of artificial neural network (ANN) among the deep learning (machine learning) methods of deep learning. It is an algorithm that performs similarity detection in the image detection method. It basically takes Web containerization, vector containerization and data containerization, which presents a methodology used to validate medical data such as EMR and PACS (see Figure 6).

**Figure 7:**
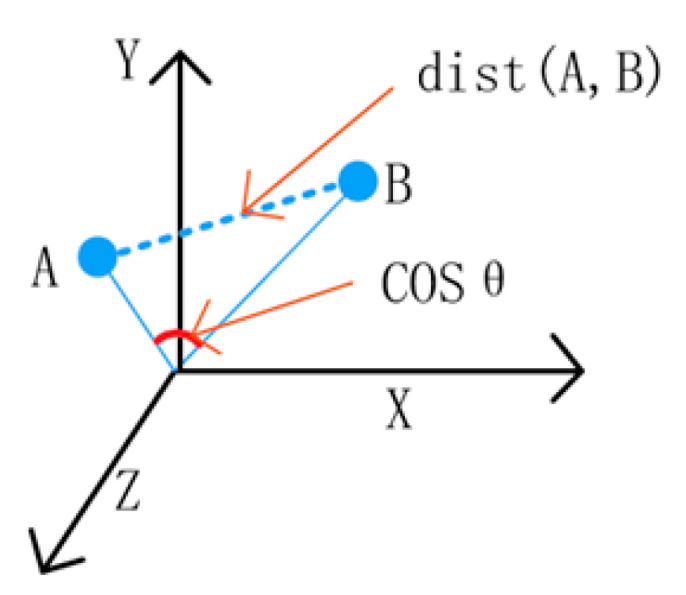
Image similarity system for extracting EMR data.

It also proposes RNN (rotational neural network), CNN (convolutional natural network), DNN (deep neural network) and DRN (dural neural network) algorithms for data analysis over time. Based on these studies, a methodology for analyzing, verifying and studying EM data, which is medical information data, is presented. Using the common CNN methodology used here, intelligent agents present a research methodology for validating data such as EMR and PACS data and performing deep learning for these data analysis as necessary. In addition, research methodologies separate database containers into web server container, expert container and database container by managing original SQL and image data volumes in separate containers to more accurately manage databases using docker container within the operational server to store the entire data in the process of collecting data for similar EM data among medical data. In addition, the calculated medical vector values can be analyzed to calculate the magnitude of the directionality according to the image similarity between the data, taking into account the shape, color, contour, etc. of the images to derive the order of similarity between images. In addition, for image calibration, the three axes of X, Y and A are performed as shown in (see Figure 7). Here, it refers to an equation in which the values of A and B are des (A, B) to obtain a value of cosine.

### 3.3. CNN intelligent Agent Cloud Architecture to Increase EMR Readings

**Figure 8:**
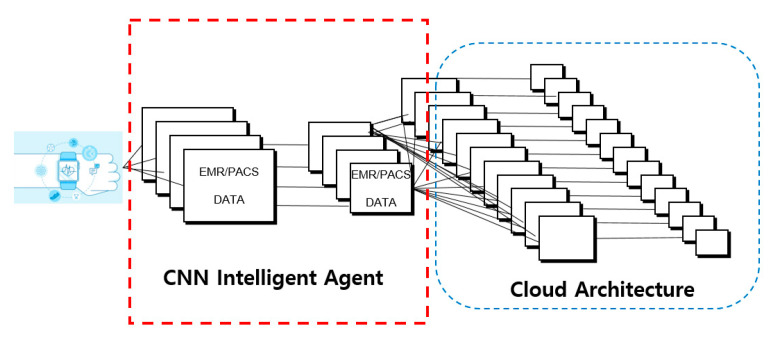
CNN intelligent agent cloud architecture to increase EMR readings.

Various related technologies such as machine learning, deep learning, imaging, natural language processing and voice recognition are applied to AI medical devices. The most active application is the convolution neural network. Convolutional neural networks are deep-learning algorithms that mimic human visual processing, especially for image recognition and classification. Convolution neural networks use a technique called convolution and pooling to recognize objects in images. Convolution creates a new image by applying a number of filters to each part of the image that emphasizes individual characteristics such as edge, contrast, etc., and pooling is aimed at reducing the size of the image made. The computer recognizes the peripheral features of the images by repeating convolution and pooling several times, climbs to a higher layer and identifies the general features of the entire image. Convolutional neural networks are especially useful for reading medical images. For example, if a medical image detects a cancer-causing nodule, the use of a convolutional neural network allows a relatively accurate distinction of nodules in a short period of time with limited image information such as X-ray images. In order to improve reading accuracy and reduce learning time, however, professional medical personnel must have sufficient annotation gold standard data. Such takes much time and money. This difficulty in securing gold standard data also hampers the performance of external verification by AI medical devices. As such, the CNN architecture did not address image data well. For data from CIFAR-10, each image consists of 32 × 32 × 3 (by side, by three color channels (red, green, blue); for one neuron in the first hidden layer, 32 × 32 × 3 = 3072 lights are required. What if the image size is much larger than this? This is how much more weight is needed; such full connectedness is a serious waste, and a large number of parameters result in overfitting. On the other hand, CNN can construct architecture in a more reasonable way. CNN’s layers usually have three dimensions: horizontal, vertical and deep (the depth represents the activation volume of this layer, not the depth of the entire neural network). For example, the CIFAR-10 image can be seen as an input activation volume with dimensions of 32 × 32 × 3 (longitudinal, depth). The last output layer will have a dimension of 1 × 1 × 10 (10 as the number of classes in CIFAR-10 data) as it will soon look at the structure in detail, but also because it makes the entire image a vector of class scores. Input data from an artificial neural network consisting only of fully connected layers are limited to one-dimensional (alignment) forms. One color photo is three-dimensional data. Multiple pictures used in batch mode are four-dimensional data. If we need to learn the fully connected neural network with photo data, we need to plane the three-dimensional photo data into one dimension. Space information will be lost in the process of flattening picture data. As a result, the lack of information due to the loss of image space information limits the extraction and learning of artificial neural networks to inefficient and accurate features. CNN is a model that can be learned while maintaining spatial information in images (see Figure 8).

### 3.4. Faster R-CNN Intelligent Agent Cloud Filter Architecture

The size of faster R-CNN can be arbitrarily set at 5 × 5 with intelligent agent architecture on the faster R-CNN filter. Next, one number is drawn for each part. Since the size of the filter is 3 × 3, there are a total of 9 parameters, and x can be outputted by one mistake via activation such as Wx+b or RELU (Wx+b) for the right W. Since the image is 10 × 10, we can see the whole by applying a filter of 5 × 5 once. In other words, if we repeat the same task by moving the filter continuously or if we move it down or sideways, we should define how far we should move it. Moreover, the amount of transfer is called stride. In this case, if the stride is given to 1, scan the entire image. Likewise, the weight W used in this process is the same. Since each step gets one value, it is possible to repeat this process to get a total of 5 × 5 output. In fact, the size of the output can be obtained by the following formula:

Total size: N (i.e., N × N × N × N × N)
Filter size: F (i.e., F × F × F × F × F)
Output size: k:=(N−F)/stride + 1 where k is an engineer.

The current example shows k = (10−5)/1 + 1 = 3. If k calculated as above is not an integer (e.g., N = 10, F = 5, stride = 5), exclude it. This output is called convolution layer. What we have done so far is that, in the end, if one filter is given one activation per filter, we will get the resulting consolidation layer. In other words, if the filter is different, the corresponding activation can be different. We can think of a new convolution layer that comes out of this. This is said to have obtained 10 convolution layers from 10 filters. In our current example, we have six 5 × 5 convolution layers because it is N = 10, F = 5. If we combine them together, we have a 5 × 5 × 10 capacity layer (or activation map). The process of obtaining activation maps is called convolution (in some situations, it is said that convolution will declare itself to get a single value from the filter). These convolutions can be done only once; depending on the situation, however, we can do several convolutions. Since activation maps are 5 × 5 × 6, we can view this as a new image and repeat the process before. If we look closely at the process of conversion, size k of the conversion layer is usually less than or equal to N. stride 1,1, F≥1.

k = (N−F(N−F)/stride + 1 ≤ N−F+1 = (N−(F−1))) ≤ N

In such cases, the 10 × 10 data will be 5 × 5 data after convolution. In other words, it will cause a significant amount of data loss. Loss of data here means a mathematical reduction in dimensions. If we ignore the amount of RGB and look at 10 × 10 and 5 × 5 only, the data expressed in 49 dimensions is reduced in 25 dimensions. In general, data loss will be inevitable because data are not embedded in 25 dimensions even before convolution. After all, our goal is to keep the form 10 × 10. The image of the 10 × 10 size is extended to 10 × 10 by adding zeroes up and down on both. For this extended 10 × 10 image, we get a 7 × 7 convolution layer. We got the same-size convolution layer as we wanted to prevent data loss.

Total size: N
Filter size: F
Stride: s
Padding size: p
Consolidation layer size after padding: k = [(N+2p)−F]/s + 1
K = N if and only if p = [(N−1)(N−1)s+(F−N)]/2

**Figure 9:**
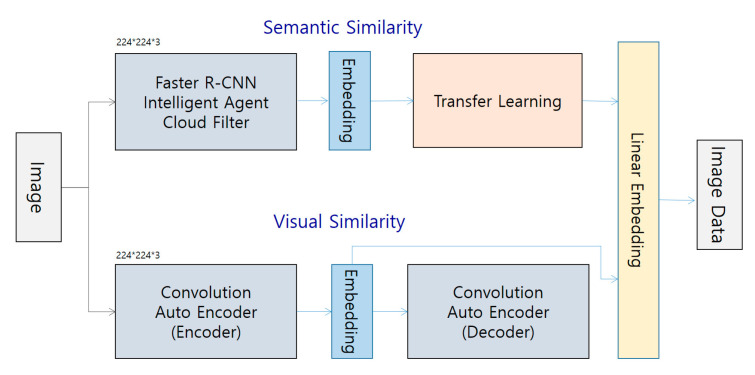
CNN intelligent agent cloud architecture to Increase EMR Readings.

If we apply the formula above, we can see that it is applied well because F = 3 = 3 = 3 = 3 = 2 = 1. While it is great to protect against losses by applying padding to cloud computing, it is also important to consider creating unnecessary parts. Since there was not enough zero attached to the original data, noise occurs other than the 10 × 10 data we originally need. Still, use padding because it is better than data loss from convolution without padding. With the faster R-CNN intelligent agent cloud filter, we want to increase the directness of extraction of EMR Data to increase the reliability of medical data. (See Figure 9) the faster R-CNN intelligent agent cloud filter has a size of 224 × 224 × 3. In addition, when image data with size 224 × 224 × 3 of the semantic similarity method and the conversion auto encoder (Encoder) method showing transfer learning through embedding is embedded, the convolution auto encoder (decoder) Data are embedded.

It shows how to do it. The architecture of this method has visual similarity. These two methods also use the methods of semantic similarity and visual similarity to take a matching methodology.

The above expression is an equation for verification of the faster R-CNN intelligent agent. This expression Hi verifies with the H of the passionately regulated adjacent matrix.

(1) $$H_{j}=\frac{A_{j}}{\sum_{k=1}^{n}A_{kj}},\;j=1,\ldots ,\;n$$

It is also designed for matrix A to have a value of zero for all of the top a diagonal components of the matrix along with each column Aj.

Moreover, the H obtained in the previous phase of the faster R-CNN intelligent agent is not the one applied to the faster R-CNN intelligent agent.

(2) $$S=H+\frac{ea^{T}}{n}$$

However, it is assumed to be 1 even if it is not all rhetoric in this procession. The faster R-CNN intelligent agent matrix H defines matrix S to make the sum of the columns 1 because the sum of the last columns in Node is 0.

This chapter presents the architecture through analysis of data libraries using core faster R-CNN filters. The necessary elements are presented in a conceptual diagram. In this chapter, records are stacked based on the filtering algorithms of RNN, faster R-CNN and structured data, which are medical data, structured data and unstructured data. Moreover, the most common concept of this concept can be divided into patterns by a central artificial intelligence processing unit (ACU). The medical information of individuals in basic condition is collected through advanced array engines. It is then defined by a verification algorithm that is more accurate or not. If by a very important algorithm it is not always thought to be accurate, but rather inaccurate, the algorithms on the faster R- CNN base make judgments. Therefore, using faster R-CNN filter, only medical data from personal information can be extracted most safely and quickly.

**Figure 10:**
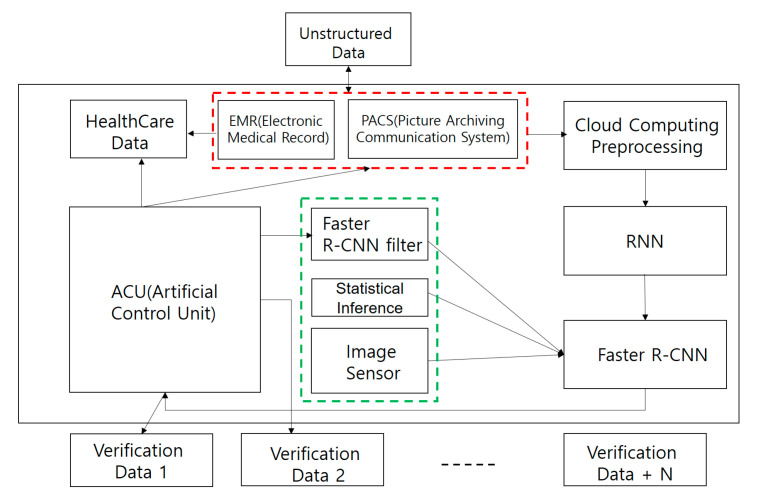
Faster R-CNN intelligent agent cloud filter architecture.

These procedures are due to the application of a secure encryption system that applies both encryption and security for personal information protection to heavy systems. In addition, through RELU (Wx + b), the medical data of patients such as images, text and videos are analyzed. As shown in (see Figure 10) the graphic uses an ACU (artificial control unit) device to verify medical data such as EMR and PACS. This concept is used to verify images in real time or in batches. In addition, prepare these data for verification with the static reference using the faster R-CNN filter. The image sensor of the medical data provides a second comparative analysis after the first screening process of the screening process. In addition, data from all databases of unstructured data and structured data are available. Moreover, all of these data were designed to be stored on a cloud computer, AWS, so that it could be verified anywhere. Continuous, non-directed learning is achieved by proven data, unstructured data are analyzed. A more accurate cognitive algorithm flow here is a key pattern in the conceptual diagram. In addition, all data are stored using personalized cloud systems and can be stored by dividing the classes of security by individuals. Image sensor processing is also a verification device for processing data such as PACS. PACS is verified using artificial intelligence verification techniques to produce verification data such as conventional X-ray, MRI and CT scans. It enables comparison through more verification and comparison functions.

When faster R-CNN is implemented, the stripe in the composite layer is mainly set to 1, and, as seen in the architecture, the size of the output image will be smaller than the size of the input image. Thus, through the composite layer, the image becomes smaller and smaller, and the information of pixels located at the edge of the image disappears. Padding is being used to solve these problems. Padding plays a role in making the input image the size of the output image equal to or similar to that of the input image by adding pixels set to the edge of the input image with a specific value. Adding pixels with a value of zero to the edge of an image is called zero-padding, and faster R-CNN-based zero-padding is used in this study.

#### 3.4.1. Faster R-CNN Algorithm Detection Technique

**Figure 11:**
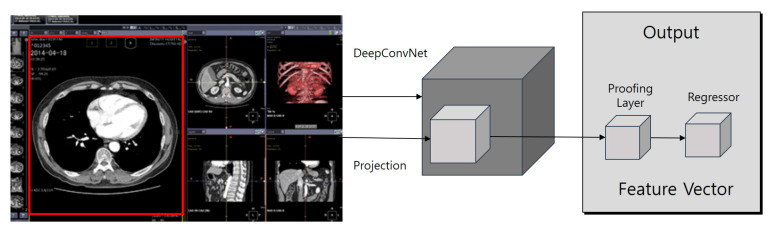
Faster R-CNN intelligent agent cloud filter architecture.

The faster R-CNN algorithm detection technique processes CNN for the entire image and extracts feature vectors for each object candidate area to produce results such as object recognition information and the location of the object’s bouncing box. It also builds data sets for EMR and PACS image deep learning among medical data. Here, the image for analysis is automatically extracted by the master R-CNN and forwarded to the analysis service. It also designs learning models that recognize characters and shape objects that make up trademark images by utilizing the faster R-CNN and deep ranking algorithms and develops learning algorithms (see Figure 11).

#### 3.4.2. EMR (PACS) Image Detection Using the Faster R-CNN Algorithm

**Figure 12:**
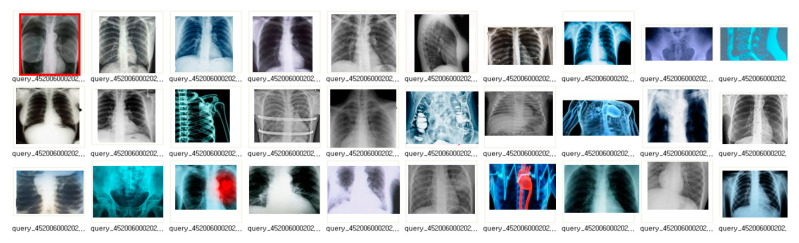
EMR (PACS) image detection using the faster R-CNN algorithm.

One thousand medical (EMR, PACS) images (approximately 120,000) were randomly extracted and compared in pairs (one target image, two comparison images). In addition, 40% of the extracted images were labeled with a negative image on the side that was not similar to the target image, and other comparative images were discarded and partially modified to label a positive image. Of the two comparative images, 30% of the extracted images were labeled as negative images, while other comparative images were discarded and partially modified to label positive images. Of the two comparative images, 20% of the extracted images were labeled as positive images and the rest as negative images (see Figure 12).

#### 3.4.3. Faster R-CNN Intelligent Agent Cloud Filter Spatial Arrangement

It is possible to reduce the parameters considerably through the faster R-CNN intelligent agent cloud filter (x, y) if any filter has been used in any location in a useful way; this filter can also be useful in other locations (x2, y2). A slice of a three-dimensional volume (two-dimensional slice cut in depth) is called a deep slice. In the example above, [55 × 55 × 96], the slice of a volume, has a size of [55 × 55]. From now on, it is limited to having the same weight and bias, common to one deep slice. Therefore, the required parameters in the example above are (slice size) × depth, so (1111113) × (96) = 34,848 different weights (weight). If we add one bias per depth, there are 96 different biases, and the total number of parameters is 34,848 + 96 = 34,944.

The internal result of one left-input volume and filter is referred to as an activity map; in the example above, the filter (11 × 11 × 3) on the input volume of (227 × 227 × 227 × 3) is S = 4, P = 0, so the size of the activity map is (55 × 15) (27–11/4 + 1 = 55). Therefore, the (96 × 55) filter has an activity map.

Let us look through the code. If the input volume is called a numpy array X, then X (light, longitude, depth) can indicate the value of each position coordinate. The activity map of the i–th dimension can be represented by X(: , : , i). If X.shape : (11,11,4) does not use zero padding (P = 0), the filter size is 5 (F = 5) and the stride is 2 (S = 2). The size of the filter is (5 × 5 × 4) and the spatial size of the output volume (by horizontal or vertical length) is (11–5)/2 +1 = 4. Therefore, the size of the output volume is (4, 4, K). As such, the output volume is V, the internal result of the input volume X (:,:5,:) (=0–4) is (4,1,1) and the internal result of the first filter (W0 below) is as follows. The two three-dimensional *s are internal operations (the sum of values multiplied by the values of the same). Therefore, the size of input volume X and output volume V of the first filter W0 is (4 × 4 × 1). The size of output volume V is (4 × 4 × 2) because there are two different kernels and two filters.

In addition, these filters can be applied as various types of filters. These 7 × 7 filters are used to verify images. Because the image has a variety of consensus processes for correction. Each image has the form of storing values on a node and verifying those values.

#### 3.4.4. Faster R-CNN Intelligent Agent Cloud Pooling Layer

It is common to insert a pooling layer in the middle of a Conv layer. What this layer does is reduce the number of parameters and the computation. Max pooling is the most common [38].

Input W1 × H1 × D1

Determine two hyperparameters.
F: Pooling filter (by size)
S: Strikes
Output W2 x H2 x D2 (pooling layer)
W2, H2 = (W1, H2 − F)/2 + 1
D2 = D1

A pooling filter does not have weight. Thus, the pooling area is not called a filter, just pooling. Max pooling is widely used in practice and is set to F = 2, S = 2. Another method of setting up is overlapping pooling of F = 3, S = 2. Remember, in the backpropagation chapter, the backward pass of max (x,y) is just like sending the gradient of the input that had the greatest value in the forward pass. Therefore, save the position of max activity during forward pass and use it for backpropagation. Because pooling layers usually reduce the size of the presentation considerably (this effect is only aided by the overfit protection effect in small datasets), the recent trend is said to be evolving gradually toward not using the pooling layer. A method of pooling filters anything smaller than fractional max-pooling 2 × 2. A random combination of filters is sizes 1 × 1, 1 × 2, 2 × 1, 2 × 2. For each forward pass, grids are randomly generated, with the test using the average of the predicted scores of several grids. The study, “Striving for Simplicity: The All Convergence Net” proposes a method that repeats only the convolution layer and does not use the pooling layer. To reduce the size of the presentation, a convolution layer with a large stride is sometimes used.

#### 3.4.5. Faster R-CNN Intelligent Agent Cloud Layer Pattern

Stacking Conv layers with filters of several smaller sizes is recommended instead of Conv layers with large receptive fields. There is an activation function between stackings. Suppose we stack three CONV layers in 3 × 3 size (of course, we put a nonlinear function between each layer). In this case, each neuron in the first CONV layer will see a 3 × 3 area of the input volume. Each neuron in the second CONV layer has the effect of seeing the 3 × 3 area of the first CONV layer and consequently the 5 × 5 area of the input volume. Similarly, each neuron in the third CONV layer will see a 3 × 3 area of the second CONV layer, equal to a 7 × 7 area of the input volume.

Simply using the 7 × 7 filter has some shortcomings, however. While three 3 × 3 conv layers can produce a good feature by inserting a nonlinear function in the middle, conv layers with 7 × 7 filters are simply linear coupling. Therefore, electrons can make better features.

Moreover, if we compare the number of parameters (all of them have K kernels), the electrons are three layers × (K × (3 × 3 × K)). = 27K^2, and the latter one-layer × (K × (7 × 7 × K)) = 49 K^2. Nonetheless, there is an advantage in doing backpropagation, i.e., the results that need to be stored in the middle use less memory in a timely manner than in the former. In some cases (especially in the old ConvNet architecture), the use of the techniques described above will increase memory usage very rapidly. For example, filtering images of 224 × 224 × 3 into 64 filters and three 3 × 3 CONV layers using stride 1 would create a total of three activation volumes with a size of [224 × 224 × 64]. This number is nearly 10 million activity values, and it uses about 72 MB of memory (per image) (for each activity and gradient). GPU usually creates bottlenecks in memory, so there is a need for some compromise with reality in this area. In practice, the first CONV layer usually finds a compromise. For example, there are cases wherein 7 × 7 filters and stride 2 (ZF net) are used in the first CONV layer. For AlexNet, use 11 × 11 filters and stride 4. LeNet, AlexNet, ZF Net, GoogleNet, VGGNet, and ResNet, etc. are available; for the description, see the following link: The following are the size and number of parameters for each layer for VGGNet: F = 3, S = 1 and P = 1 for Conv layers and F = 2, S = 2 and P = 0 for Pool layers.

### 3.5. Deep-rank Algorithm of Faster R-CNN

The deep-rank algorithm is completed in step 2. First, we must open a source classification algorithm. The semantic similarity is learned through fine tuning using the previously classified trademark image data using a existing pre-learned algorithm. When looking for recommended systems, health care data are verified using methods such as collaborative filtering, content-based filtering and hybrid. It is then verified with a library implemented by matrix authorization. However, health care data can be used to verify that memory usage increases rapidly or learning speed slows down as learning data increases. It sets up the overall recommendation system in service because it is not flexible to utilize a variety of information rather than a single value of the user’s ID of health care data. The deepak recommendation system has a first-stage candidate model that selects hundreds of candidates to recommend to users, and a second-tier rank model that calculates how many of those candidates will interest users. Considering the reason for dividing into two stages, users are often too long to calculate the score of interest for all articles, so they quickly pick the candidates to recommend and then calculate and rearrange the ranking scores. The learning principle is similar to matrix authorization (MF). MF decomposes into user vectors and item vectors when there is a user item matrix, and again predicts the score with the user vectors and item vectors by dot product operation. Various health care data information is learned through the neural network to generate user vectors. As a result, user vectors and content vectors are the same principle of dot product computation. We must apply a language model that predicts the next image from the health care image to the previous images. The first comparison of health care data are similar in predicting the next image based on 100 images. So, we learn a language model. In addition, learn using negative sampling and binary cross entropy loss. An important part of the fast-real service is used to predict similar vector searches. Although the calculation burden is low due to negative sampling when learning, it will take a long time to calculate all of the user vectors and the entire text when forecasting. Dot-product operations are used as calculations to find similarities, which can be replaced by similar vector searches. Therefore, similar vector searches of health care data can be found very quickly through learning and these parts can be less of an implementation burden with a well-implemented library. Unlike the candidate model, it learns both user information and writing information by concatting them. Moreover, the final result is from the neural network layer rather than the dot product, which more accurately predicts preference. It is then verified with a regression model that indicates the degree of value of health care data. Due to the high complexity of the overall recommendation system, the project is divided step-by-step. In addition, the recommendation system is applied to the service by implementing the candidate model, and the next step is to verify the health care data with the rank model.

### 3.6. Time Variable of Faster R-CNN

Next, we must verify health care data by applying time variables. Time variables are a method of predictive-based machine learning. Time variables are mainly used in strengthening learning problems. Moreover, use a combination of the Monte Carlo method and dynamic planning method. It features the Monte Carlo method, in which learning required for time variables is conducted by sampling the environment according to certain policies, and the dynamic planning method of obtaining current estimates using past learned estimates. The time variable learning algorithm is used as a time variable learning model for health care data. The learning of time variables is verified with the following predictions involved. In time variable prediction-based instruction learning, only the values observed by health care data are taught. In other words, when predictions are made and results are observed, the forecasting mechanisms are modified to better meet the observed results. Time-variable algorithms modify forecasts to fit not only the observed results, but also other forecasts for the future. This process is called bootstrap. Take the following example: Algorithms for comparing health care data and time variable learning algorithms both optimize certain cost functions related to the error of predictions for certain probability variables. In addition, time variable algorithms also verify real-time and placement programs of health care data in particular.

### 3.7. Error Rate of Faster R-CNN

For the actual error rate of health care data, the phenomenon of learning closer to the distribution of learning samples than the actual distribution is called overfitting, so how to avoid overfitting is called regularization. This phenomenon also occurred in fashion-MNIST datasets where the previous health care data were depreciated. If we change the model’s structure or hyperparameters during an experiment, some combinations have found that the model is not accurate when using a testing dataset compared to the learning data. Apply learning and generalization errors. Errors that come when learning datasets of health care data are used for learning errors and generalization errors validate the expected errors when applying the model to virtual streams from which additional data are extracted from the underlying data distribution. Often generalized errors are estimated by applying the model to the test set. We calculate the learning error and generalized error rate using squared loss function used in linear regression or cross-entropy loss function used in softmax reaction among loss function discussed previously. If the input of health care data is discreet, it can work well by looking at many samples. However, it is highly likely that the model will not work well in real life if the data are real values or more than we want. Furthermore, we have only a limited amount of memory to store the model. In addition, simple classification problems are applied to lower error rates. Locate a d-dimensional polynomial to estimate y, if given learning data consisting of scalar data characteristics (feature x) to reduce the error rate of health care data and scale y for it.

y^ = ∑i = 0dxiwi(3)

where wi means the weight parameter of the model and bias is x0 = 1, so w0. For simplicity, use squared loss as in the case of linear stress.

The polynomial function of high dimension is more complex than the polynomial function of low dimension. The reason is that when the dimension is higher, it has more parameters and a wider range of choices for the model function. Therefore, if the same learning dataset is used, learning errors for higher-level polynomial functions will be lower than those of lower-level polynomial functions. It also applies the general correlation of model complexity and errors when the learning dataset is fixed. If the model is too simple for the data, underfitting occurs and overfitting occurs when the model is too complex. Choosing the appropriate complexity of the model for the data solves the issues of overfitting and underfitting.

### 3.8. Design Patterns (State Pattern) of the Faster R-CNN Intelligent Agent Cloud Architecture

State means state. Object states, such as the stationary, falling and rising states of elevators, also vary depending on the situation. State patterns are used to treat the same behavior differently depending on the state of the object. When several states exist for such an object (e.g., stop, rise, fall), programming without using a pattern is done using an if statement or a switch statement. If a new condition (e.g., door open, door closed) occurs, however, the program must be modified again.

In such cases, state patterns allow for different states to be handled (upState, stopState, downState) in a way that encapsulates the state of an object and causes it to be referenced by a state interface, as illustrated below. Thus, modification of the raw code can be minimized at the time of change (add new state), and maintenance can be made easier. Based on the status of the faster R-CNN intelligent agent cloud architecture, it draws design patterns for it. These design patterns show the status values of the layers on the faster R-CNN intelligent cloud layer pattern. The agent also contains structural details that allow real-time viewing of status information of EMR data and shows status information accordingly. Furthermore, shows the design pattern of the faster R-CNN intelligent agent cloud. This defines an agent. An agent is an object that processes all image data. Clients also receive values from these agents. In addition, the faster R-CNN intelligent agent cloud agent has a function that can call up all faster R-CNNs. Similarly, use the spatial arrangement, parameter sharing. Moreover, finally, it defines an object called filter.

## 4. Experiments and Results

### 4.1. Experimental Environment

In this study, the CUDA SDK benchmark program is performed on GPGPU–SIM developed based on simple scalar, a reliable simulator, in order to accurately measure the performance of GPGPU in order to take performance data of artificial intelligence (see Table 2).

(1) Performance of GPU

Key factors that determine GPU performance include hardware components and internal network components. The GPGPU–SIM takes this into account, largely consisting of a part of a system simulator that runs a benchmark program based on hardware components and a part of a network simulator that connects each core within the GPU.

(2) System components of the CNN system simulator

- NVIDIA’s QuadroFX5800
- QudroFX5800
- NVIDIA SLI Multi-OS
- Multiple Quadro GPUs
- Workstations in virtualized environments (HP ML-500)
- Booksim structure
- Network environment internal network net
- Network topology: Mesh

### 4.2. Comparison of Faster R-CNN Intelligent Agent Cloud Architecture with General EMR Performance

#### 4.2.1. Sensitivity Performance Experiment

This study cites experimental data using artificial intelligence methodologies. This implements the architectural design methodology using the faster R-CNN intelligent agent cloud filter Spatial Arrangement. Some of the excessive source code was opened.

Moreover, some of the CNN intelligent agent cloud filter spatial source code has also been opened. The faster R-CNN intelligent agent cloud pooling layer was also designed and implemented. The core methodology was analyzed by including a broad range of areas from various perspectives in relation to artificial intelligence technology. In addition, Stanford AI index distinguished the key detailed indicators by their activity and technical performance. In addition, the amount of activity is composed of academe, industry, open source software and public interest, and the technical performance is analyzed by visual, natural language understanding, proof of cleanup and resolution of satisfactory problems. Specificity analyses the amount of activity by dividing the technical performance into visual, natural language understanding, proof of organization and problem solving of fulfillment potential. Visual technology is also analyzed by organizing metrics with object recognition and visual data questions and answers.

Sensitivity also analyses natural language comprehension skills by constructing indicators through sentence analysis, machine translation, question and answer and speech recognition. Additionally, there are no separate sub-metric metrics for proof of cleanup and solvable problem resolution.

**Figure 13:**
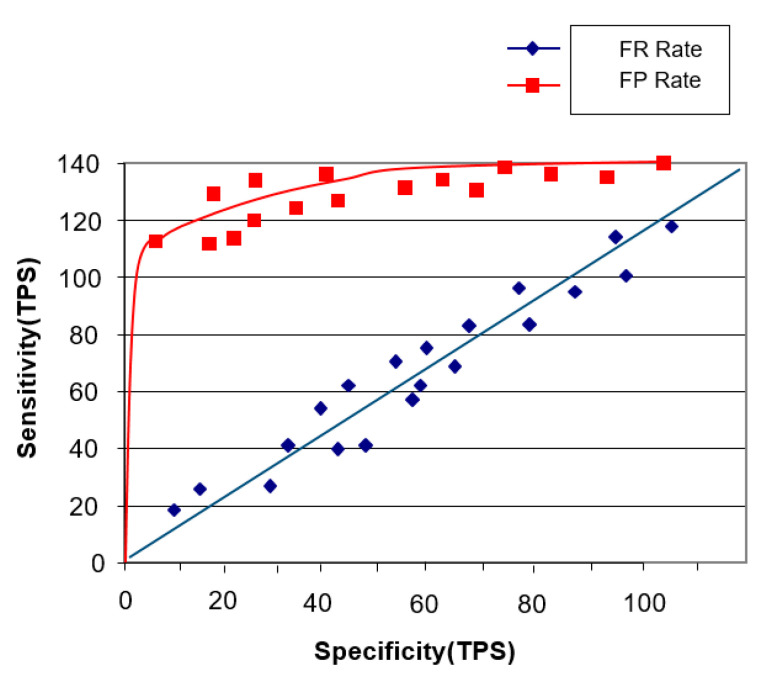
Models not covered by the faster R-CNN intelligent agent cloud architecture.

Thus, the aforesaid concept of artificial intelligence without the faster R-CNN intelligent agent cloud architecture can be considered to be generally less sensitive (see Figure 13).

**Figure 14:**
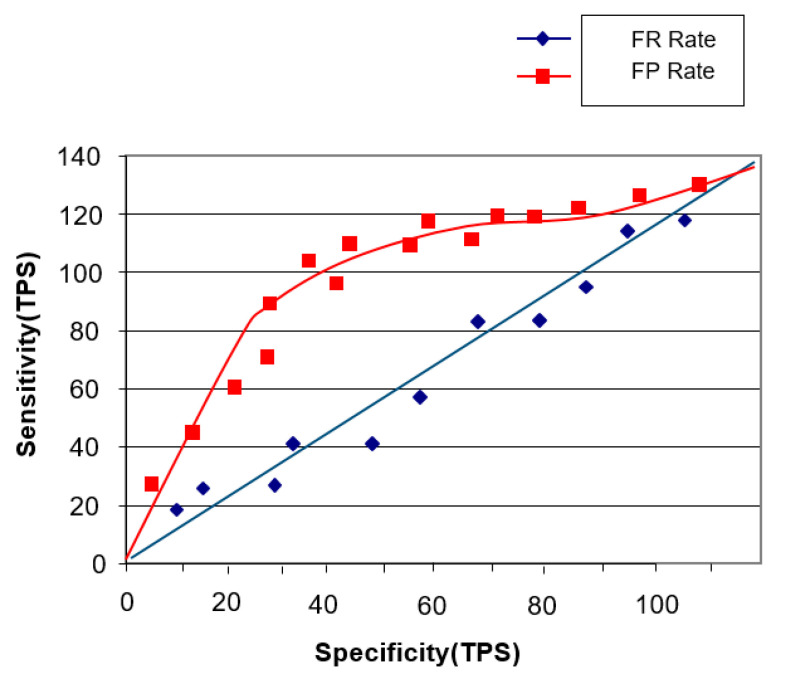
Model with faster R-CNN intelligent agent cloud architecture.

Furthermore, if we look at the fact that the faster R-CNN intelligent agent cloud architecture is one of the concepts of artificial intelligence, we can see that sensitivity is over 140. The holdout method used above also calculates the point image for the generalized accuracy of the model. In some applications, the confidence interval near this estimate is more useful and necessary and point estimation may be sensitive to a particular set of training/test sets (see Figure 14).

When converting the characteristics of images in (see Table 2) into vectors, it was confirmed that the accuracy increased by acting as a plus factor if the two aspects were considered together compared to when the meaningful and visual information of the images were taken individually, as shown in the model comparison table. In addition, ConvNet, which determines the semantic similarity, was able to improve its performance through continuous additional data learning in the form of a combination of multiple faster R-CNN algorithms and additional layers and when measuring the accuracy of the convolutional automotive modeler, it is lower than other image models.

#### 4.2.2. Image Staticity Performance Test

##### Image Staticity Performance Test Process

**Figure 15:**
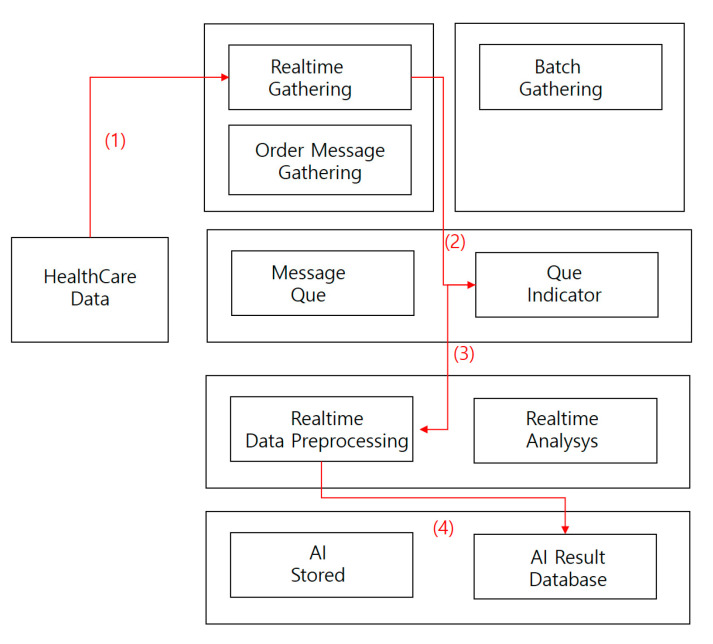
Image staticity performance test process.

The process of measuring performance by processing medical image data was as follows (see Figure 15).

(1) Collect medical imaging data in real time.
(2) Store medical image data collected in real-time processing directly in a message queue.
(3) Store collected data in a real-time preprocessor.
(4) Save AI results database data.

##### Image Staticity Performance Test Condition

The procedure for organizing verification data for medical imaging data was as follows;

- 1000 medical images (approximately 120,000 cases) were randomly extracted and 4 images (two target images and four comparative images) were compared.
- 40% of the extracted images were labeled as negative images of two comparative images that were not similar to the target image and other comparative images were discarded as positive images by transforming some of the target images.
- 50% of the extracted images were labeled as negative images, while other comparative images were discarded and partially modified to label positive images.
- 20% of the extracted images had positive images of the two comparative images that were similar to the target image the rest were labeled as negative images.
- If the target and comparison images could not be determined to be similar, four random images were extracted from the medical image (repeated until deemed similar).
- 40% of the extracted images were positive images of two medical images compared to the target image, and the remaining images were labeled as negative images.
- If it was not possible to judge that the target image was similar to the target image, the data set was constructed with the current target image.
- Using the above method, 2000 data sets consisting of query images, positive images and negative images were generated by odd numbers of video data to complete the final verification data by combining the results in a majority vote.

##### Image Staticity Performance Test Simulation

**Figure 16:**
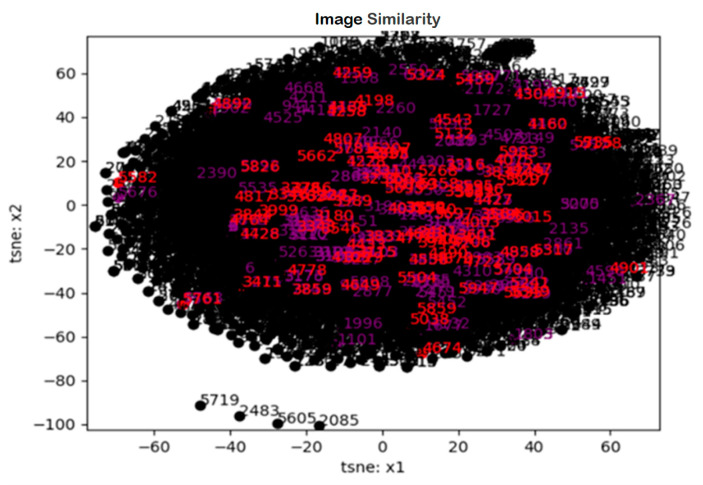
Image staticity performance test result (Image similarity).

Visualization of results verified the similarity of medical image data. The distribution plots of tsne: x1 and tsne: x2 were distributed very evenly in the middle. Moreover, from the point in the center, it appears to be distributed as positive and negative values. We could see that this was very similar (see Figure 16).

##### Image Staticity Performance Test Result

In the case of CovNet (Semantic) +, the similarity was 93.51%, showing the most similar medical image comparison. In addition, F-source had a value of 0.93472. Moreover, in the case of CovNet (Semantic), the similarity was 91.65% and the similarity was moderate. The F-source was 0.91234 and for DenseNet201, the similarity was 87.45%, showing the lowest similarity. Moreover, the F-source showed a value of 0.89232 (see Table 3).

## 5. Discussion

This study is based on the fast R-CNN algorithm and how accurately the existing medical image data are verified. Hence, we are doing the necessary research here. Various verification methodologies were applied to verify the results. The first was the sensitivity performance expert test. The experimental environment was constructed by upgrading from the existing system for this project.

We created a verification environment by constructing GPGPUs such as NVIDIA’s Quadro FX5800, NVIDIA SLI Multi-OS, etc. servers, middleware servers and operating servers and conducted a study with four physical units. The sensitivity performance experience also showed a sensitivity of about 140%. Finally, the method of testing data was to collect medical image data in order to process it in real time, as well as to store real-time-collected medical image data directly in the message queue. It stores collected data in real-time preprocessors and stores AI result database data. This resulted in data with a similarity of 93.51% a very high figure. This research is intended to extract and research more data for medical imaging in the future by introducing more cloud systems, etc. Moreover based on these research data we will continue with an optimization study. In the future, we are planning to study cancer data among these health care data. Cancer is causing pain to many people all over the world. Lung cancer, liver cancer and pancreatic cancer all lead to many deaths in humans. By analyzing cancer data using deep-learning technologies, we plan to make data sets that can be used for early verification and prevention and verify them by prediction algorithm. We also want to utilize and compare these MRI and CT data to improve accuracy. We will continue on to this research.

## 6. Conclusions

This study aimed to verify the similarity of medical data by applying fast-R-CNN techniques. This is because this technique minimizes errors in reading medical image data by analyzing vast amounts of medical data. Hence, we designed the architecture for image similarity. It analyses medical image data by dividing it using web, and data container methods. These data also have X, Y, Z values. We also designed an algorithm to verify these values against COS data. The consistency of the data was verified by dividing them using semantic similarity verification. We used visual similarity verification to verify these data as accurately as possible. In the case of ACU an algorithm that makes it possible all algorithms are controlled. As such, control money algorithms led to fast R-CNN algorithm patterns. This algorithm analyzed the vector values of images through deep ConvNet. Finally, 12,000 medical imaging data were verified. One thousand of 120,000 medical images (approximately 120,000 cases) were extracted as side effects, and the results were taken by comparing each of the four. In addition, 40% of the extracted images were labeled as negative images by comparing two images that were not similar to the target image. Other comparative images are discarded.

The target image was partially modified to be labeled as positive image; 50% of the extracted images were labeled as negative images. The other comparative images were discarded to partially modify the target image labeled as a positive image. Of the two comparative images, 20% of the extracted images were labeled as positive, and the rest as negative. If the target and comparison images were similar, four random pages were re-extracted from the medical imaging image. Forty percent of the extracted images were labeled as positive. The remaining were labeled as negative images—similar to the target image, of the two medical image comparison images. Subsequently, if it was not possible to judge that the target image was similar to the current target image, the data set was composed of the comparison image. The final method was to complete 2000 data sets consisting of a querying image, a positive image and a negative image; the final result was to be verified with odd number of images. As a result of the experiment, four units were physically constructed and tested. Sensitivity performance experience also showed a sensitivity of about 140%. Finally, the method of testing data was to collect medical image data in order to process it in real time and to store the medical image data that was collected in real time directly in the message queue. It stored collected data in real-time preprocessors and stored AI result database data. As a result, the similarity CovNet (Semantic) + had 93.51% of the data, CovNet (Semantic) had 91.65% and DenseNet201 had 87.45% of the similarity. These similarities allow for the convergence of 93.51% of medical imaging data. In the future, we will do research to further increase the similarity of these medical image data.

## Figures and Tables

**Table 1:** Smart health care for hardware, software and service.

Items	Hardware	Software	Service
Purpose	Hardware systems such as robots during artificial intelligence research	Software technology key to the study of artificial intelligence	Personalized models found
Related research	Wearable devices, parts, devices, reagents, etc.	Providing medical health care content, communication network platform, medical information, exercise information, etc.	Genes, medical diagnosis services, genetic information
Speed Measurements	High	Middle	Slow
	Robot systems for healthy strengthening	Personalized, integrated medical device services	Hardware and software mixed service required
Middleware	None	Middleware required	Needed
Technical technology	Blood sugar, blood pressure, ECG, activity measurement, chemical analysis, body fat analysis, medical sensors, field testing devices, band-necked implants	WebnisApp, nutrition management app, personal health care app	Personal health examination services, personal health records management systems and health care services for the elderly

(Source: Health Care Time, 2019) [5].

**Table 2:** Experimental environment equipment list.

Sortation	Standard	Quantity	Relative Height
Development server	Xeon E5-2620 v4 2.1Ghz 32 vCPU (8 core 16 thread 4 CPU) 512 GB RAM SAS HDD 0.8 TB * 2, SAS HDD 1.2 T * 6, ATA SSD 0.8 TB * 6	4	Windows Server NT
Middleware server	CORBA 3.0	1	Linux Server (Redhat)
Operating server	Xeon E5-2620 v4 2.1Ghz 32 vCPU (8 core 16 thread 4 CPU) 512 GB RAM SAS HDD 0.8 TB * 2, SAS HDD 1.2 T * 6, ATA SSD 0.8 TB * 6	4	Windows Server NT
GPGPU system	Multiple Quadro GPUs	4	Windows Server NT

**Table 3:** Image staticity performance test result.

Test Model	Modify Contents	Accuracy	F-Score
CovNet (Semantic)+	transfer learning	93.51%	0.93472
CovNet (Semantic)	transfer learning	91.65%	0.91234
DenseNet201	transfer learning	87.45%	0.89232

## Abbreviations

EMR	Electronic Medical Record
PACS	Picture Archiving Communications System
OCS	Order Communication System
EHR	Electronic Health Record
MRI	Magnetic Resonance Imaging
ACU	Artificial control unit
AI	Artificial intelligence
ANN	Artificial neural network
CNN	Convolved convolutional neural network
DNN	deep neural network
DRN	Dural neural network
DRNN	deeply reliant neural network
EHR	Electric health record
FDA	Food and drug administration
IPFS	Interplanetary file system
ML	Machine learning
MRI	Magnetic resonance imaging
OCS	Order communication system)
OODBMS	Object database is a database management system
PACS	Picture archiving and communication system
R-CNN	Region-based convolved convolutional neural network
RNN	Rotating neural network
